# Sepsis among Adults Admitted to Intensive Care Unit of a Tertiary Care Centre

**DOI:** 10.31729/jnma.8439

**Published:** 2024-02-29

**Authors:** Kundu Shrestha, Sagun Ram Vaidya, Keny Shrestha, Surendra Man Shrestha, Prabhat Rawal, Shova Dangol

**Affiliations:** 1 Department of Anesthesiology and Critical Care, Nepal Armed Police Force Hospital, Balambu, Kathmandu, Nepal; 2 Department of Anesthesiology and Critical Care, Nepal Medical College, Jorpati, Kathmandu, Nepal; 3 Department of General Practice, Royal Cornwall Hospital, NHS Trust, Treliske, UK

**Keywords:** *intensive care units*, *prevalence*, *sepsis*

## Abstract

**Introduction:**

Sepsis a syndrome that starts with an infection, causes organ dysfunction, and leads to death is a global health issue in critically ill patients. While its epidemiology is well-known in high-income countries, it is poorly understood in low- and middle-income countries, including Nepal. This study aimed to find out the prevalence of sepsis among adults admitted to the intensive care unit of a tertiary care centre.

**Methods:**

This descriptive cross-sectional study was conducted among adults admitted to the intensive care unit after obtaining ethical approval from the Ethical Review Board. Data was collected from 11 January 2022 and 29 December 2022 from hospital records. A convenience sampling method was used. The point estimate was calculated at a 95% Confidence Interval.

**Results:**

Among 195 patients, the prevalence of sepsis was seen in 74 (37.95%) (31.14-44.76, 95% Confidence Interval). Septic patients had a median age of 44 (interquartile range: 33.75-60.25) years. A total of 40 (54.05%) were male. A total of 28 (37.84%) septic patients were identified with >2 diagnoses, while 9 (12.16%) had >2 comorbidities.

**Conclusions:**

The prevalence of sepsis among adult patients admitted to the intensive care unit was higher as compared to other studies done in other international studies.

## INTRODUCTION

Sepsis, whether asymptomatic or fulminant, is associated with high morbidity and mortality.^1^ Systematic reviews revealed an annual global prevalence of 13-300 and 11 per 100,000 people for severe sepsis and septic shock, respectively.^2^ Even though sepsis is declining due to advances in supportive care and guidelines, it remains the leading cause of non-cardiac death for critically ill patients.^3^ A staggering 8-26% of deaths result from these conditions, along with increased treatment costs.^4^

Sepsis in intensive care unit (ICU) patients is well documented in developed countries,^4^ but there is limited data in low- and middle-income countries. Nevertheless, these data help increase awareness of sepsis, identify more effective preventive and therapeutic interventions, and guide resource allocation.^5^ However, epidemiology of sepsis in Nepal is limited and may not be representative of the epidemiology of ICU patients.

This study aimed to find out the prevalence of sepsis among adults admitted to the intensive care unit of a tertiary care centre.

## METHODS

This descriptive cross-sectional study was conducted in the Department of Anesthesiology and Critical Care, Nepal Armed Police Force Hospital, Balambu, Kathmandu, Nepal. Data was collected from 11 January 2022 and 29 December 2022. Ethical approval was taken from the Ethical Review Board of the Nepal Health Research Council (Reference number: 898). Patients over 16 years old admitted to the ICU were included in the study. Individuals with incomplete data were excluded. A convenience sampling method was used. The sample size was calculated by using the following formula:


$$\begin{array}{ll} \text{n} & ={\text{Z}}^{2}\times \frac{\text{p}\times \text{q}}{{\text{e}}^{\text{2}}}\\ & =1.96^{2}\times \frac{0.50\times (1-0.50)}{0.08^{2}}\\ & =150 \end{array}$$

Where,

n = minimum required sample size
Z = 1.96 at 95 % Confidence Interval (CI)
p = prevalence taken as 50% for maximum sample size
q = 1-p
e = margin of error, 8%

Hence, the minimum sample size required was 150. However, the final sample size taken was 195.

Sepsis is defined as a dysregulated host response to infection leading to life-threatening organ dysfunction whereas, septic shock is considered as a subset of sepsis where underlying circulatory, cellular, and metabolic abnormalities lead to a high risk of mortality.^5^ Following the diagnosis of sepsis using standard guidelines, the sequential organ failure assessment (SOFA) score and daily evaluation of organ function were done with organ failure being determined if the SOFA sub-score for a specific organ was greater than 2. Previously, both clinical and microbiologic infections were reported daily, with infection being defined according to the criteria set by the International Sepsis Forum.^5^

Demographic details (age and gender) and clinical information (diagnosis, comorbidities, length of ICU stay, mechanical ventilation, and outcome) of all patients in the ICU were obtained from hospital records. Data from the blood culture reports were documented in the case records of the patients.

Data were entered in Microsoft Excel 2010 and analyzed using IBM SPSS Statistics version 17.0. The point estimate was calculated at a 95% CI.

## RESULTS

Among 195 patients, the prevalence of sepsis was seen in 74 (37.95%) (31.14-44.76, 95% CI). The median age of septic patients was 44 years, with an interquartile range of 33.75-60.25 years. A total of 20 (27.03%) were between the ages of 31-40 years (Table 1).

**Table 1:** Distribution of patients based on age group and gender (n = 195).

Age (years)	Gender		
	Female (n = 34)	Male (n = 40)	Total
11-20	1 (2.94)	-	1 (1.35)
21-30	3 (8.82)	6 (15)	9 (12.16)
31-40	8 (23.53)	12 (30)	20 (27.03)
41-50	5 (14.71)	8 (20)	13 (17.57)
51-60	6 (17.65)	7 (17.50)	13 (17.57)
61-70	8 (23.53)	5 (12.50)	13 (17.57)
71-80	2 (5.88)	2 (5)	4 (5.41)
81-90	1 (2.94)	-	1 (1.35)

There were 28 (37.84%) septic patients with ≥2 additionaldiagnoses, and 9 (12.16%) septic patients had ≥2 comorbidities.Among the septic patients, the most frequent diagnosis waspneumonia 21 (26.58%), closely followed by acute kidneyinjury 14 (17.72%) (Table 2).

**Table 2:** Additional diagnoses among septic patients (n = 79).

Diagnosis	n (%)
Pneumonia	21 (26.58)
Acute kidney injury	14 (17.72)
Acute respiratory distress syndrome	6 (7.59)
Chronic obstructive pulmonary disease	6 (7.59)
Hyponatremia	6 (7.59)
Acute pancreatitis	4 (5.06)
Decompensated liver disease	4 (5.06)
Gastrointestinal injury	4 (5.06)
Cancer	2 (2.53)
Poisoning	2 (2.53)
Diabetic ketoacidosis	2 (2.53)
*Others	8 (10.13)

Infrequent incidences of electrocution,pulmonary abscess, fracture, and seizure were also reported. A total of 16 (41.03%) had diabetes mellitus (Table 3).

**Table 3:** Comorbidities among patients with sepsis (n = 39).

Comorbidities	n (%)
Diabetes mellitus	16 (41.03)
Hypertension	10 (25.64)
Hypothyroidism	8 (20.51)
Atrial fibrillation	2 (5.13)
Chronic kidney disease	2 (5.13)
Rheumatoid arthritis	1 (2.56)

A total of 34 (45.95%) needed ventilator support while mortality was seen among 28 (37.84%). The average length of stay in the ICU was 3.42±1.85 days.

## DISCUSSION

Among 195 patients, the prevalence of sepsis was seen in 74 (37.95%). Another study conducted in Ethiopia reported an overall prevalence of sepsis and septic shock at 26.5%,^3^ while in Egypt, it was 24.8%.^4^ In a Colombian city, the prevalence was found to be 44%,^6^ and in Jordan, it was 23.3%.^7^ The differences in the prevalence were due to differences in setting, differences in the diagnostic criteria used and population.

The presence of infection and organ dysfunction was consistent with the findings of other significant studies (24.8-40%), including the Worldwide Data from the Intensive Care Over Nations Audit and Sepsis Occurrence in Acutely Ill Patients (SOAP).^4,5,8^ According to the 2017 Global Burden of Disease Study, 48.9 million cases of sepsis occurred worldwide, with 11 million deaths, accounting for 19.7% of all global deaths.^9^ This study underlines the immense impact of sepsis on modern ICUs. Despite a global variation in sepsis rates, note that East and Southeast Asia have particularly high rates, confirming a high disease burden there.^5^ We believe this disparity is related to variations in ICU admissions and causes among enrolled patients since our patients carried substantial infection burdens.^4^

In the current study, the majority of the patients with sepsis were males (54.05%), and the overall median (interquartile range) age was 44 (33.75-60.25) years. Unlike in this study (47.85±15.81 years), a higher mean age (51.62±18.62 years) was reported for septic patients in another study, despite a higher rate of sepsis among men (72%).^4^ In previous study septic patients averaged 54.5 years of age, indicating that sepsis occurs more frequently in older individuals.^6^ The variation in outcomes could be attributed to the inclusion of a larger study population. This study found that most patients aged 51-70 years (35.14%) were septic, partially agreeing with another study which posited that patients with severe sepsis are mostly aged 55-64 years.^4^ A study also found a direct correlation between advanced age and severe sepsis, with a marked increase among the elderly population.^10^

In this study, the average length of stay in the ICU was 3.42±1.85 days, which was substantially lower than the findings in another study (12.72±7.55 days).^4^ In this study, 45.95% of septic patients required mechanical ventilation, which is notably lower than the 59% identified by another study.^4^ Nonetheless, we conclude that the longer ICU stay, with more frequent use of medical equipment, will eventually increase hospital costs.

Compared to the other study (54.9%), this study had a lower mortality rate among septic patients at (37.84%).^4^ Similarly, an ICU study in 2011 from 150 hospitals across 16 countries found sepsis mortality rates to be high (44.5%) in Asia.^11^ Our findings, however, were in line with those of other similar studies which found that 23% of deaths in ICUs occurred among 1184 adults recruited from 59 units.^12^ Sepsis-related mortality in the ICU is likely due to increased co-existence of the underlying conditions and complications, including pulmonary and heart disease.^4^ Additionally, delayed treatment and poor compliance with sepsis bundles are probably the most important causes of high mortality in patients with severe sepsis in ICUs.^13^ Among ICUs in Brazil and India severe sepsis had a mortality rate of 55.7%, and 64.6% respectively.^14,15^ Differences in intensive care facilities and treatments, as well as the number of patients included in each report, may contribute to these differences.^4^

In this study, diabetes mellitus (41.03%) was the most commonly associated chronic disease that was found in 41.18% of non-survivors, followed by hypertension, which was found in 23.53% of non-survivors. This comes as no surprise considering the high and growing prevalence of diabetes mellitus in Nepal and the devastating multisystem effects it has.^10^

There are a few limitations of our study. The current study is limited by a small sample size and being conducted at a single centre, potentially impacting the generalizability of the findings. Furthermore, precise information on all subtypes of microorganisms, their resistance patterns, and the appropriateness of antimicrobial coverage was not collected.

## CONCLUSIONS

This prevalence of sepsis among adult patients in the intensive care unit was found to be higher as compared to other studies done in other international studies. Further research with larger sample sizes and multicenter studies in diverse settings is recommended to enhance the understanding of sepsis prevalence, risk factors, and outcomes, enabling more effective strategies for sepsis prevention and management in intensive care units.
